# Deletion of IL-33R (ST2) Abrogates Resistance to EAE in BALB/C Mice by Enhancing Polarization of APC to Inflammatory Phenotype

**DOI:** 10.1371/journal.pone.0045225

**Published:** 2012-09-18

**Authors:** Marija Milovanovic, Vladislav Volarevic, Biljana Ljujic, Gordana Radosavljevic, Ivan Jovanovic, Nebojsa Arsenijevic, Miodrag L. Lukic

**Affiliations:** Center for Molecular Medicine and Stem Cell Research, Faculty of Medical Sciences, University of Kragujevac, Kragujevac, Serbia; French National Centre for Scientific Research, France

## Abstract

The administration of interleukin 33 and deletion of IL-33 receptor, ST2 molecule, affects the induction of autoimmunity in different experimental models of human autoimmune diseases. The aim of this study was to analyze the effect of ST2 deletion on the induction of experimental autoimmune encephalomyelitis (EAE) in resistant BALB/c mice. Mice were immunized with MOG_35–55_ peptide or disease was induced by passive transfer of encephalitogenic singenic cells and EAE was clinically and histologically evaluated. Expression of intracellular inflammatory cytokines, markers of activation and chemokine receptors on lymphoid tissue and CNS infiltrating mononuclear cells was analyzed by flow cytometry. We report here that deletion of ST2^−/−^ molecule abrogates resistance of BALB/c mice to EAE induction based on clinical and histopathological findings. Brain and spinal cord infiltrates of ST2^−/−^ mice had significantly higher number of CD4^+^ T lymphocytes containing inflammatory cytokines compared to BALB/c WT mice. Adoptive transfer of ST2^−/−^ primed lymphocytes induced clinical signs of the disease in ST2^−/−^ as well as in WT mice. MOG_35–55_ restimulated ST2^−/−^ CD4^+^ cells as well as *ex vivo* analyzed lymph node cells had higher expression of T-bet and IL-17, IFN-γ, TNF-α and GM-CSF in comparison with WT CD4^+^ cells. ST2^−/−^ mice had higher percentages of CD4^+^ cells expressing chemokine receptors important for migration to CNS in comparison with WT CD4^+^ cells. Draining lymph nodes of ST2^−/−^ mice contained higher percentage of CD11c^+^CD11b^+^CD8^−^ cells containing inflammatory cytokines IL-6 and IL-12 with higher expression of activation markers. Transfer of ST2^−/−^ but not WT dendritic cells induced EAE in MOG_35–55_ immunized WT mice. Our results indicate that ST2 deficiency attenuates inherent resistance of BALB/c mice to EAE induction by enhancing differentiation of proinflammatory antigen presenting cells and consecutive differentiation of encephalitogenic T cells in the draining lymph node rather than affecting their action in the target tissue.

## Introduction

Multiple sclerosis is the inflammatory disease of the CNS, characterized by inflammatory lesions, demyelination and axonal loss [1]. Experimental autoimmune encephalomyelitis (EAE) is the experimental model of multiple sclerosis induced in susceptible animals by active immunization with myelin antigens mixed with adjuvant. T lymphocytes activated by encephalitogen in the periphery differentiate in inflammatory helper T cells able to pass blood-brain barrier where they recognize their cognate target antigen and initiate an inflammatory cascade leading to tissue damage. EAE also can be induced by passive transfer of myelin reactive population of CD4^+^ T helper cells. However T helper differentiation toward inflammatory phenotype with encephalitogenic potential depends on the function of antigen presenting cells.

ST2 molecule is a member of the IL-1 receptor family [2] that exists in two forms: a transmembrane full length form (ST2L) or a soluble form (sST2) due to differential splicing of ST2 mRNA [3]. Soluble ST2 can serve as decoy receptor. Full length ST2 is expressed by many hematopoietic cells, monocytes, dendritic cells, macrophages NK and NKT cells, mast cells, and granulocytes and selectively by murine Th2 [4], and human Th2 cells [4]. ST2 is reported as a marker of effector Th2 cells that enhances Th2 response [5]. Natural ligand for ST2L (IL-33Rα-chain) is IL-33, a member of the IL-1 family of cytokines. IL-33 can act as classical cytokine that binds to ST2L and IL-1R accessory protein (IL-1RAp), activate NFkB and MAPK [6], [7]. IL-33 can also stimulate innate type 2 immune cells, natural helper cells from adipose tissues and lung [8]–[10], nuocytes from mesenteric lymph nodes and spleen [9], and innate helper 2 cells from various tissues [11] to produce large amounts of IL-5, IL-6, IL-13 and GM-CSF [8], [9]. IL-33 can enhance LPS mediated TNF and IL-6 production by macrophages [12], [13] and induce IFN-γ production in iNKT and NK cells [14]. IL-33 was firstly described as important mediator of allergic diseases [15] and resistance to parasite infection [16]. IL-33 also has proinflammatory role in collagen induced arthritis [17], [18] and ulcerative colitis and experimental Th1/Th2 driven enteritis [19] inducing production of IL-5, IL-6, and IL-17 in mucosal immune cells. On the other hand we have shown that in other models of Th1 cell mediated inflammation such as multiple low dose streptozotocin induced diabetes type I [20] and Con A induced hepatitis [21] ST2 deletion enhanced disease development.

The highest levels of IL-33 in the body are found in the brain and spinal cord [22] and this cytokine is mainly produced by astrocytes [23]. IL-33 receptor is expressed mainly on microglial cells and its stimulation with IL-33 enhances production of inflammatory cytokines IL-1β and TNF-α, as well as nitric oxide and also enhances phagocytosis in microglial cells [23]. The other report indicates increased level of IL-33 in the inflamed brain tissue, in mice infected with Theiler’s murine encephalomyelitis virus as well as in C57BL/6 mice after EAE induction [24] and suggests IL-33 activity in the CNS as the factor in CNS inflammatory diseases through induction of type 2 innate responses. Expression of ST2 molecule is upregulated in the brains of mice infected with T. gondii and ST2 deficiency increases susceptibility to this infection and increases levels of iNOS, IFN-γ and TNF-α in the CNS of infected mice [25]. Previous report indicates that lack of IL-33 does not affect EAE development in susceptible C57BL/6 mice [26], while very recent report suggests key role of IL-33 in the pathogenesis of EAE [27] based on findings that anti-IL-33 treatment significantly reduced the incidence and severity of EAE, whereas IL-33 administration worsens the disease course, when administered before the onset of clinical symptoms. However our recent work clearly demonstrates that IL-33 given therapeutically ameliorates EAE in susceptible C57BL/6 mice and that IL-33R deletion facilitate EAE induction in resistant BALB/c mice [28].

We now demonstrate that in resistant BALB/c mice ST2 deletion affects inductive rather than effector phase of the disease. Analysis of the cellular events in the immunized lymph nodes indicates that in the absence of IL-33/ST2 signaling dendritic cells after stimulation with encephalitogen acquire proinflammatory phenotype, while T cells acquire chemokine receptors required for their recruitment into CNS. Finally encephalitogenic ST2^−/−^ cells transferred EAE to naïve mice while ST2^−/−^ dendritic cells induced the disease in MOG_35–55_ immunized resistant, singenic hosts.

## Materials and Methods

### Animals

Female 6 to 8 week old C57BL/6, BALB/c WT and ST2^−/−^ BALB/c mice were used throughout this study for the induction of EAE and adoptive transfer experiment. Target disruption of ST2 gene was performed in BALB/c embryonic stem cells as previously described by Townsend et al [29]. Mice were maintained in our maintained in our animal facilities in a temperature-controlled environment with a 12-hour light/12-hour dark cycle and were administered standard laboratory food and water *ad libitum.* All experiments were approved by and conducted in accordance with the Guidelines of the Animal Ethics Committee of Faculty of Medicine University of Kragujevac, Serbia.

### Induction and Scoring of EAE

EAE was actively induced by subcutaneous administration of 200 µL suspension at 2 sites over the hind flanks. The suspension consisted of 300 µg MOG_35–55_ peptide (Sigma Aldrich, Germany) in 100 µL of PBS, emulsified with 100 µL complete Freund`s adjuvant (Sigma Aldrich, Germany) with 0.7 mg heat-inactivated *Mycobacterium tuberculosis* (strain H37 RA; Difco Laboratories, Detroit, MI). Each mouse was immediately thereafter, injected intraperitonealy and 48 hours later with 300 ng pertussis toxin (List Biological Laboratories, Campbell, USA) in 100 µL 0.9% NaCl. Clinical signs of EAE were assessed daily by the following scoring system: grade 0, no signs; grade 1, paralyzed tail; grade 2, ataxic; grade 2.5, one hind leg paralyzed; grade 3, both hind legs paralyzed; grade 3.5, 3 legs paralyzed; grade 4, both hind legs completely paralyzed and beginning front limb paralysis; grade 5, moribund as previously described [30]. Mice were monitored daily with fluid administration and mashed chow on the base of cages for all mice displaying a clinical score of 3.

### Adoptive Transfer of EAE

Passive induction of EAE was done as described previously [31]. BALB/c WT and ST2^−/−^ BALB/c mice were immunized with 300 µg MOG_35–55_ emulsified in CFA. Nine days after immunization, cells were isolated from regional, popliteal lymph nodes and 1×10^7^ cells/mL were cultured with 40 µM MOG_35–55_ peptide in media containing RPMI 1640 (Sigma-Aldrich) supplemented with 10% heat-inactivated fetal bovine serum (Sigma-Aldrich, Germany), 100 U/mL penicillin (Sigma-Aldrich), 100 µg/mL streptomycin (Sigma-Aldrich), 4 mM L-glutamine (Invitrogen), and 50 µM 2-mercaptoethanol (Sigma-Aldrich) for 72 hours at 37°C with 5% CO_2_. After 72 hours, cells were harvested, resuspended at 1×10^7^ cells in 250 µL PBS and intravenously injected into recipient mice.

### Histology

Brains and spinal cords were removed and fixed in 4% buffered formalin fixative overnight. Paraffin wax embedded sections (5 mm) were stained with hematoxylin & eosin. The level of infiltration was graded using the following score [32]: 0, no inflammatory cells; 1, a few scattered inflammatory cells; 2, organization of inflammatory infiltrates into perivascular cuffs; 3, extensive perivascular cuffing with extension into adjacent subarachnoid space and CNS parenchyma and 4, extensive perivascular cuffing with increasing subarachnoid and parenchymal inflammation.

### Isolation of Mononuclear Cells from Lymph Nodes, Spleen, Peripheral Blood and Central Nervous System

Mice were perfused with PBS and lymph nodes, spleen, brain, and spinal cord were carefully removed. Lymph nodes or spleens were minced in RPMI 1640 (Sigma Aldrich) and forced gently through 40-mm cell-strainer nylon mesh (Falcon) using a sterile syringe plunger and centrifuged at 400 g for 5 min. Pellet from lymph nodes was resuspended in RPMI 1640 containing 10% FBS. Pelleted cells isolated from spleen were incubated with 2 ml ammonium chloride/Tris-chloride (pH 7.2) (erythrocyte lysing buffer) at room temperature for 5 min, then supplemented with 1 ml FBS, centrifuged subsequently at 400 g 5 min and resuspended in RPMI 1640 with 10% (vol/vol) FBS. Peripheral blood cells were isolated using BD Pharm Lyse™ lysing solution (BD Biosciences) according to the manufacturer instructions.

The mononuclear cells from CNS were isolated at the peak of the disease as descibred previously [33]. Briefly, the brains and spinal cords were separately homogenized in RPMI 1640 with 10% FBS and 1 mg/ml collagenase type I (Sigma-Aldrich) and incubated at 37°C for 60 min. After digestion the tissue was passed through a 70 mm mesh, pelleted, resuspended in 10 ml 30% Percoll (Sigma-Aldrich), overlaid onto 5 ml 70% Percoll and centrifuged at 390 g for 20 min. The myelin layer was removed and the mononuclear cells accumulated in the intermediate phase were collected, washed twice in PBS and resuspended in medium. Total cell numbers were determined by counting on a hemocytometer, and viability was assessed by trypan blue exclusion.

**Figure 1:**
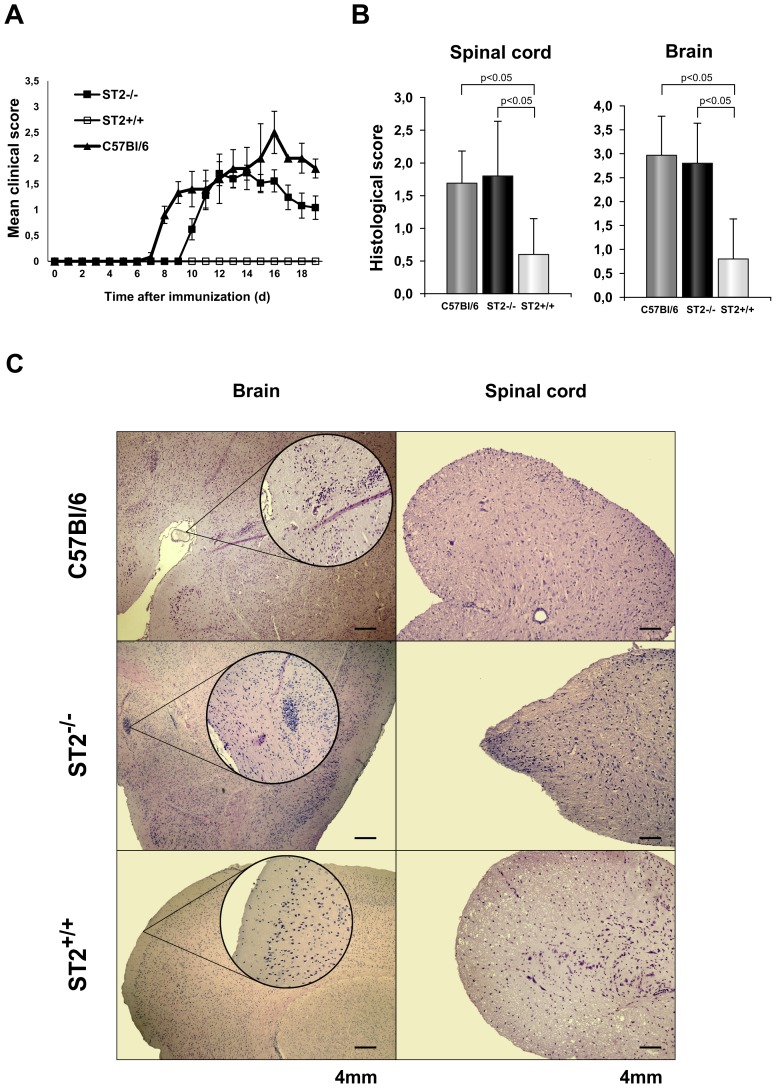
ST2^−/−^ BALB/c and C57BL/6 mice are susceptible while BALB/c mice are resistant to EAE. C57BL/6, BALB/c ST2^−/−^ and BALB/c WT mice were immunized with MOG_35–55_/CFA and were checked daily for (A) clinical signs of EAE. Data are representative of two independent experiments, twelve mice per group (means ± SEM). At the peak of the disease spinal cord and brain tissues were fixed, sectioned, and stained with hematoxylin and eosin. Histological scores were calculated from a total of 5 sections per group (B). Representative spinal cord and brain tissue sections are presented; magnification is 40 X (C). Statistical significance was tested by Student’s t-test.

### Flow Cytometry

For cytofluorometry following antibodies were used CD4, CD8, CD11b, CD11c, CD45, CD25, CD69, CCR6, CXCR3, CXCR5, F4/80, CD86, MHCII, T-bet, GATA3, Foxp3, IL-17, IFN-γ, TNF-α and GM-CSF with conjugated fluorochromes (BD Biosciences). Antibodies were incubated with cells for 30 min at 4°C and then cells were analyzed. For flow cytometric analysis of cytokines, cells were stimulated with PMA and ionomycin (P/I) plus GolgiStop (BD Biosciences) for 4–6 h, followed by cell-surface marker staining, then were fixed and permeabilized using Cytofix/Cytoperm Kit (BD Biosciences) before staining with the anti-cytokine Abs. Data were acquired using an FACSCalibur (BD Biosciences) and analyzed with FlowJo software (Tree Star).

### Cytometric Bead Assay (CBA) and ELISA

For analysis of cytokine production, 1×10^7^/ ml cells isolated from regional lymph node were cultured with MOG_35–55_ peptide at concentration 40 µM for 72 hours. Supernatant was removed and incubated with IL-6, IL-17 and GM-CSF mouse cytokine capture beads (Mouse flex sets; BD Biosciences) for 2 hours at room temperature. Data were acquired using the FACSCalibur (BD Biosciences) and analyzed with CellQuest software (BD Biosciences). Concentrations of TNF-α and IFN-γ in supernatants were measured by enzyme-linked immunosorbent assay using ELISA kits (R&D Systems) according to manufacturer’s instructions.

Dendritic cells were isolated from spleen of BALB/c WT and ST2^−/−^ BALB/c mice 10 days after immunization, using Dynabeads Mouse DC Enrichment Kit (Invitrogen) according to manufacturer’s instructions and stimulated with Pam3CysSerLys4 (1 µg/ml) for 24 hours. Levels of IL-6, IL-23 and IL-10 in supernatants were obtained using ELISA kits (R&D Systems).

**Figure 2:**
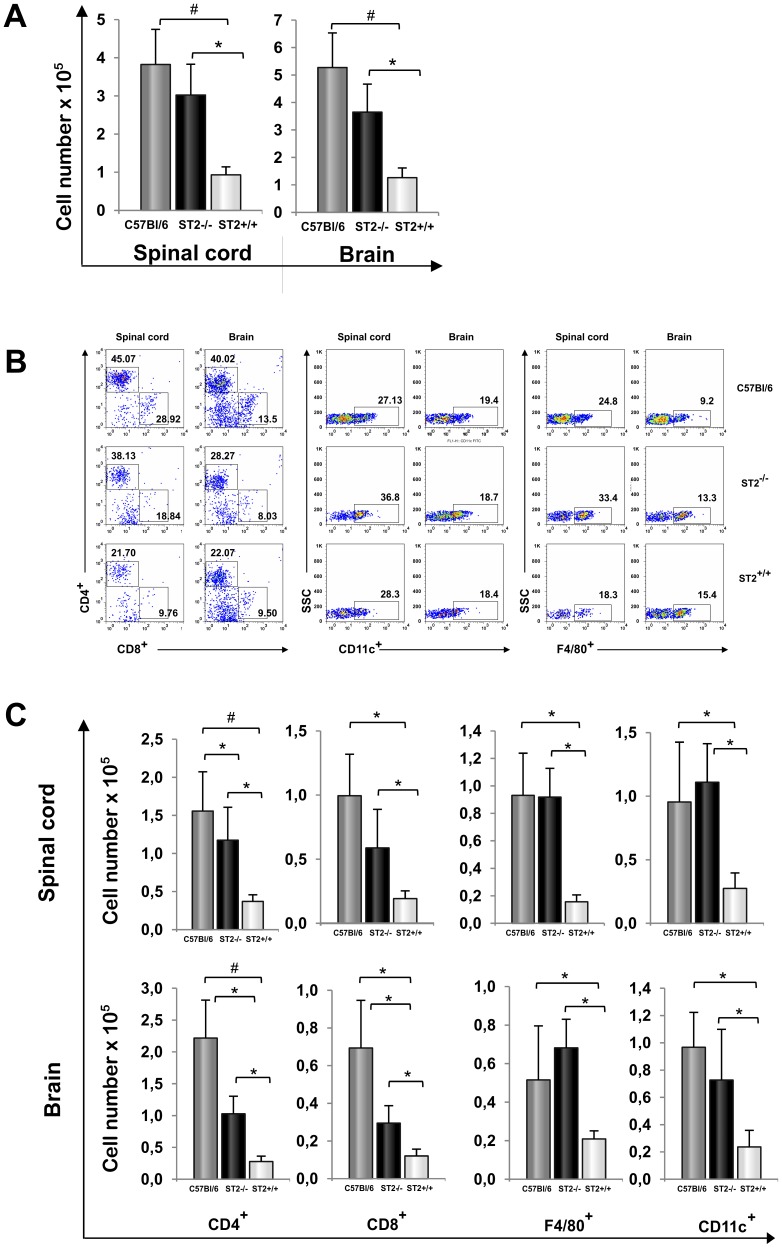
ST2^−/−^ BALB/c mice have similar infiltration in CNS as C57BL/6 mice. Fourteen days after immunization mononuclear cells were isolated from spinal cords and brains and counted (A). Pooled mononuclear cells isolated from brains and spinal cords (three per group, four samples in group, 12 mice per group) were used for flow cytometric analysis of percentages (B) and total cell numbers (C) of CD4^+^, CD8^+^, CD11c^+^, and F4/80^+^ cells. Absolute numbers were calculated as the product of the average number of CNS mononuclear cells harvested per mouse pooled from brains and spinal cords. The data are from representative experiment (mean+SD *P<0.05 and #P<0.005). Statistical significance was tested by Student’s t-test.

### MTT Assay for T cell Proliferation

For *in vitro* proliferation of CD4^+^ cells, draining lymph nodes were collected from ST2^−/−^ BALB/c and BALB/c WT mice 8 days after immunization and CD4^+^ T cells were purified with Dynal Mouse CD4 Cell Negative Isolation Kit (Invitrogen), while dendritic cells were isolated from spleens of these mice using Dynabeads Mouse DC Enrichment Kit (Invitrogen). CD4^+^ cells (2×10^5^) were cocultured in a 96-well plate with splenic dendritic cells (4×10^3^) in complete RPMI 1640 medium only, or with MOG_35–55_ (10 µg/ml) for 72 hours. The CD4^+^ cells without dendritic cells that received complete RPMI-1640 medium with MOG_35–55_ (10 µg/ml) were regarded as the control group. After 72 hours incubation at 37°C in a 5% humidified CO_2_ atmosphere, 15 µL of 3–2 (4, 5-dimethylthiazol-2-yl) 2, 5-diphenyltetrazolium bromide (MTT) (5 mg/mL) (Sigma-Aldrich, Germany) was added to each well and cultured for another 4 hours. Crystals were dissolved with 150 µL dimethyl sulfoxide and 20 µL glycine buffer after pelleting and removing media. The plates were shaken for 10 minutes, and then the absorbance was quantified at a wavelength of 595 nm using a microplate reader (Zenyth 3100 Multi-Mode-Detektor, Anthos, Austria). The results were expressed as a percentage of proliferation (S) (S = (optical density with dendritic cells – optical density of dendritic cells only)/(optical density without dendritic cells) x 100 of each animal.

**Figure 3:**
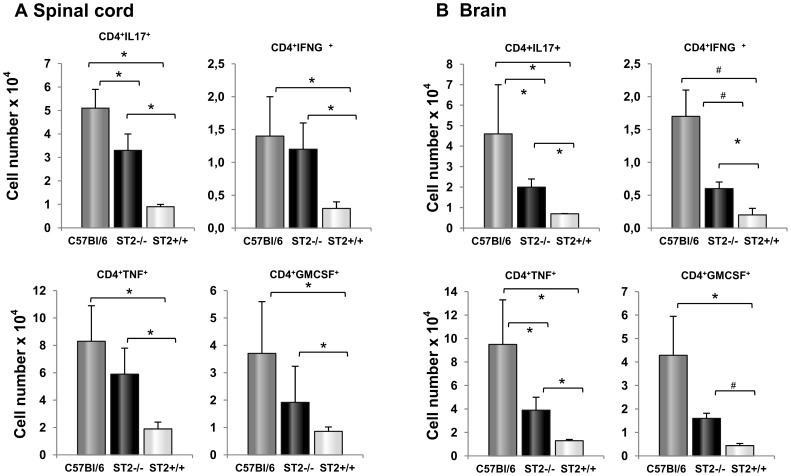
Spinal cord and brain infiltrates from ST2^−/−^ BALB/c mice contain inflammatory T helper lymphocytes. Mononuclear cells were isolated from spinal cord (A) and brain tissue (B) of mice killed at the peak of the disease and analyzed by flow cytometry after intracellular staining for inflammatory cytokines. The total numbers of CD4^+^ cells containing IL-17, IFN-γ, TNF-α and GM-CSF are calculated as the product of the average number of CNS mononuclear cells harvested per mouse from pooled brains and spinal cords. Presented data are from representative experiment, 12 mice in each group (means+SD). Statistical significance was tested by Student’s t-test.

### Dendritic Cells Adoptive Transfer Experiment

BALB/c WT and ST2^−/−^ BALB/c mice were immunized with MOG_35–55_ peptide emulsified in CFA and 4 and 8 days after immunization dendritic cells were isolated from spleens and draining lymph nodes using Dynabeads Mouse DC Enrichment Kit (Invitrogen). WT and ST2^−/−^ dendritic cells were injected intravenously to BALB/c WT EAE mice (2×10^6^ cells per mouse) on day 4 and 8 after immunization. EAE development was monitored daily using the same clinical scoring system as described above.

### Statistical Analysis

All statistics were carried out using SPSS 13.0 for Windows software. Results were analyzed using the Student’s t and Mann Whitney test. Data in this study were expressed as the mean+SD or mean+SEM. Values of p<0.05 were considered significant.

## Results

### ST2 Deletion Leads to Susceptibility to EAE and Development of CNS Infiltrates

Mouse strains differ in their susceptibility to EAE induction. C57BL/6 mice are susceptible while BALB/c mice are resistant [34]. However, as evaluated by clinical and histological score ST2^−/−^ BALB/c mice are also found to develop the disease indicating that IL-33/ST2 axis may be involved in different strain susceptibility to EAE induction. To study the role of IL-33/ST2 axis on the resistance to EAE in BALB/c mice we compared clinical and histological characteristics of the disease development in C57BL/6, BALB/c WT and ST2^−/−^ BALB/c mice (Fig. 1). Absence of clinical signs of EAE in BALB/c WT mice (Fig. 1A) is consistent with histologically minimal leukocyte infiltration in the CNS of these mice in contrast to the significant perivascular and submeningeal infiltration in areas of the spinal cord and brain of ST2^−/−^ BALB/c and C57BL/6 mice (Fig. 1B). Infiltration in CNS of ST2^−/−^ mice, expressed by histological score (Fig. 1B) and total cell number (Fig. 2A), was similar with CNS infiltration of susceptible C57BL/6 mice and significantly higher in comparison with infiltrates of resistant BALB/c WT mice.

**Table 1:** EAE after passive transfer of ST2^−/−^ MOG_35–55_ stimulated cells.

Mouse strain^a^	Incidence	Disease onset	Mean maximal score^b^	Mean cumulative score^b^
ST2^−/−^	10/10 (100%)	9,70±2,16	3,00±1,04	13,20±5,90
ST2^+/+^	8/10 (80%)	10,37±2,92	3,00±1,03	9,10±6,53

a Passive transfer of ST2^−/−^ MOG_35–55_ specific mononuclear cells was performed in ST2^+/+^ and ST2^−/−^ mice two experiments/group, with five mice/experiment, giving a minimum total, n = 10 mice per group.

b Mean cumulative score is calculated as the mean of cumulative daily scores for each mouse. Mean maximal score is calculated as the mean of most severe EAE score for each mouse. The mean cumulative and mean maximal scores include all mice within a group, even those not afflicted by EAE.

**Figure 4:**
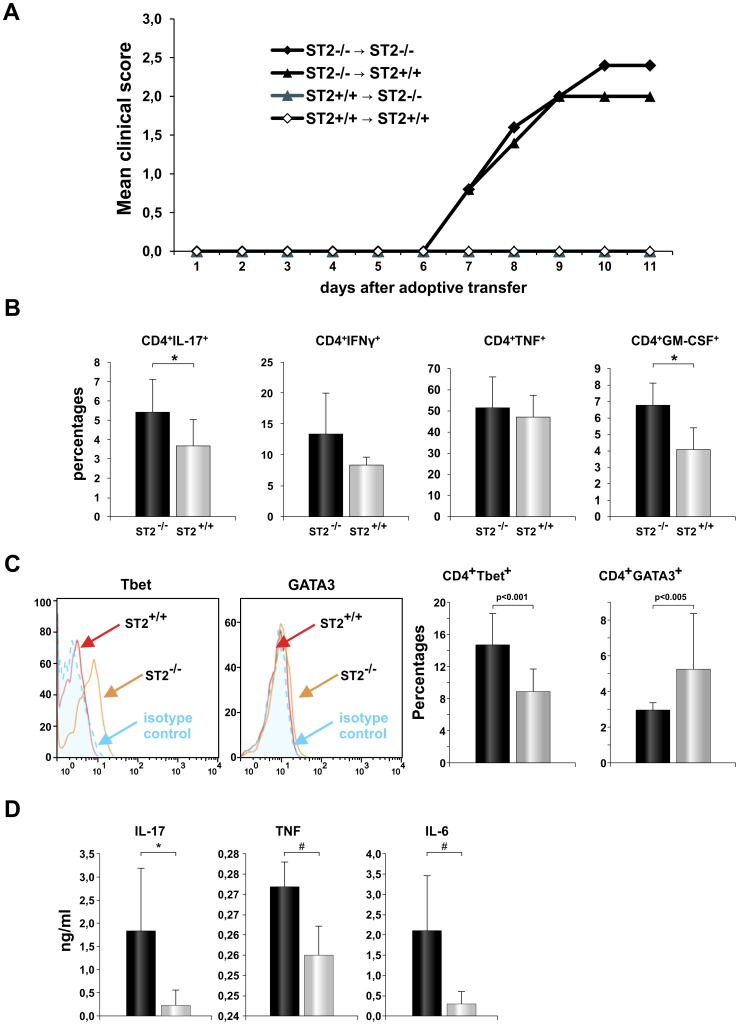
Primed ST2^−/−^ T cells induce EAE in WT and ST2^−/−^ recipient. The donor cells were isolated from popliteal lymph nodes 9 days after foot pad immunization of ST2^−/−^ BALB/c and BALB/c WT mice with MOG/CFA emulsion. Donor cells were restimulated with MOG_35–55_ peptide (50 µM) for 72 hours and 1×10^7^ were transfer to ST2^−/−^ and WT BALB/c mice. Mice were observed every day after injection for disease onset and clinically scored. Data are presented as the mean of two independent experiments with 5 animals in each group in each experiment (n = 10 in both groups * P<0.05) (A). Lymph node's mononuclear cells harvested from donor mice and restimulated *in vitro* with MOG_35–55_ peptide (50 µM) for 72 hours are analyzed for expression of inflammatory cytokines (B) and markers of Th1 and Th2 differentiation (C). Data are presented as the mean+SD from representative experiment with 10 animals in each group. Histograms and mean values presented in the graphs are gated on CD4^+^ cells (C). Levels of proinflammatory cytokines were measured in cell culture supernatants by ELISA and CBA. Data are presented as the mean+SD of two independent experiments, 10 animals per group (D). Statistical significance was tested by Student’s t-test.

In order to further characterize the event at the level of the target tissue we compared cellular make up of the mononuclear cells in the three groups of mice (Fig. 2) BALB/c ST2^−/−^ mice and susceptible C57BL/6 mice showed significantly higher infiltration of inflammatory cells subpopulations (CD4^+^ and CD8^+^ T lymphocytes, F4/80^+^ and CD11c^+^ cells) then in BALB/c WT mice. In comparison with infiltrations in C57BL/6, ST2^−/−^ mice had lower total number of CD4^+^ and CD8^+^ cells in the brain infiltrates (Fig. 2C) and lower total number of CD4^+^ cells in spinal cord (Fig. 2C), while number percentages of F4/80^+^ macrophages and CD11c^+^ dendritic cells (Fig. 2B) and total cell number of CD11c^+^ (Fig. 2C) was higher in the spinal cords of ST2^−/−^ mice. Interestingly we did not find any difference between the groups regarding T regulatory cell populations: CD4^+^CD25^+^FoxP3^+^; CD4^+^FoxP3^+^ and CD8^+^FoxP3^+^ (data not shown).

Further we assessed the effects of ST2 deletion on percentage and total number of proinflammatory T cells in the nervous tissue. We therefore quantified IL-17, IFN-γ, TNF-α and GM-CSF producing CD4^+^ T cells invading the CNS at the peak of the disease. Flow cytometric analysis of the brain and spinal cord mononuclear cells showed that IFN-γ, GM-CSF and TNF-α containing cells invading the brain as well as spinal cord of ST2^−/−^ BALB/c mice, whereas these cells were nearly absent in BALB/c WT mice (Fig. 3). However the number and percentage of IL-17 producing cells were significantly higher in both spinal cord (Fig. 4A) and brain (Fig. 4B) of C57BL/6 mice compared to ST2^−/−^ BALB/c mice. Nevertheless, we observed that all three populations of encephalitogenic T cells (IFN-γ^+^, GM-CSF^+^ and IL-17^+^) reached the brain of ST2^−/−^ BALB/c mice.

**Figure 5:**
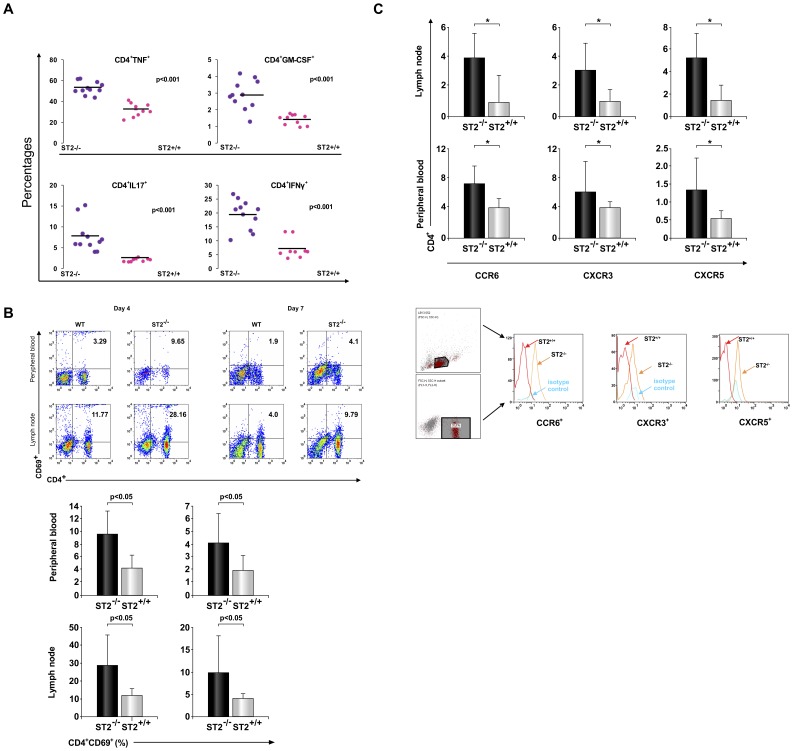
ST2 deletion leads to the induction of inflammatory T helper lymphocytes in draining lymph nodes. Mononuclear cells from draining lymph nodes of immunized ST2^−/−^ BALB/c and BALB/c WT mice were isolated on day 9. (A) Flow cytometry was used to evaluate IL-17, IFN-γ, TNF-α, GM-CSF and IL-10 containing CD4^+^ T cells. Data are presented as individual percentages of all mice, 10 mice per group. (B) Expression of activation marker CD69 on cells isolated from lymph nodes and peripheral blood 4 and 7 days after immunization was evaluated by flow cytometry. Plots are gated on lymphocyte population. Mean values of CD4^+^CD69^+^ cells are obtained from two experiments with 16 mice per group (C) Flow cytometric analysis of expression of chemokine receptors CCR6, CXCR3 and CXCR5 on CD4^+^ lymphocytes was performed on cells isolated from lymph nodes (4 days after immunization) and peripheral blood (7 days after immunization). Percentages of double positive cells CD4^+^CCR6^+^, CD4^+^CXCR3^+^ and CD4^+^CXCR5^+^ are presented (upper panel) and chemokine receptor expression in CD4^+^ population of lymph node cells (lower panel). Data are presented as the mean+SD, 16 mice per group (*P<0.05). Statistical significance was tested by Student’s t-test.

### Immune Lymphocytes Derived from ST2^−/−^ BALB/c Mice Have Inflammatory Phenotypes and Transfer EAE to Resistant BALB/c Mice

Passive transfer experiments were done in order to determine whether deletion of ST2 molecule in target tissue is essential for EAE in BALB/c mice. ST2^−/−^ BALB/c and BALB/c WT mice were immunized with MOG_35–55_ and 9 days later lymphocytes from draining lymph nodes were isolated and restimulated with MOG_35–55_ *in vitro* for 72 hours and 10^7^ WT and ST2^−/−^ cells were transferred into WT or ST2^−/−^ recipients. Only ST2^−/−^ lymphocyte were able to initiate EAE development, BALB/c WT mice developed to some degree milder EAE after transfer of ST2^−/−^ lymphocytes as well as ST2^−/−^ recipient (Fig. 4A) but there was no significant difference in EAE between two strains of mice based on clinical score, incidence and day of onset, mean maximal score and mean cumulative score (Table 1). Lymphocytes from BALB/c WT mice could not induce EAE (Fig. 4A). Further, we analyzed MOG_35–55_ specific CD4^+^ cells before transfer and found higher percentages of CD4^+^ cells containing proinflammatory cytokines IL-17, IFN-γ, TNF-α and GM-CSF among cells isolated from ST2^−/−^ mice (Fig. 4B). The difference was observed regarding IL-17 (p<0.05) and GM-CSF (p<0.05) containing CD4^+^ cells. Higher percentage of CD4^+^ cells containing proinflammatory cytokines in ST2^−/−^ mice is in correlation with higher percentage of these cells containing T-bet transcriptional factor and lower percentages of cells containing GATA-3 (Fig. 4C). Similar results were obtained by ELISA of cell culture media of lymphocytes challenged with MOG_35–55_ *in vitro*, which demonstrated that IL-17, IL-6 and TNF-α production was significantly higher in ST2^−/−^ lymphocytes in comparison with WT lymphocytes (Fig. 4D).

**Figure 6:**
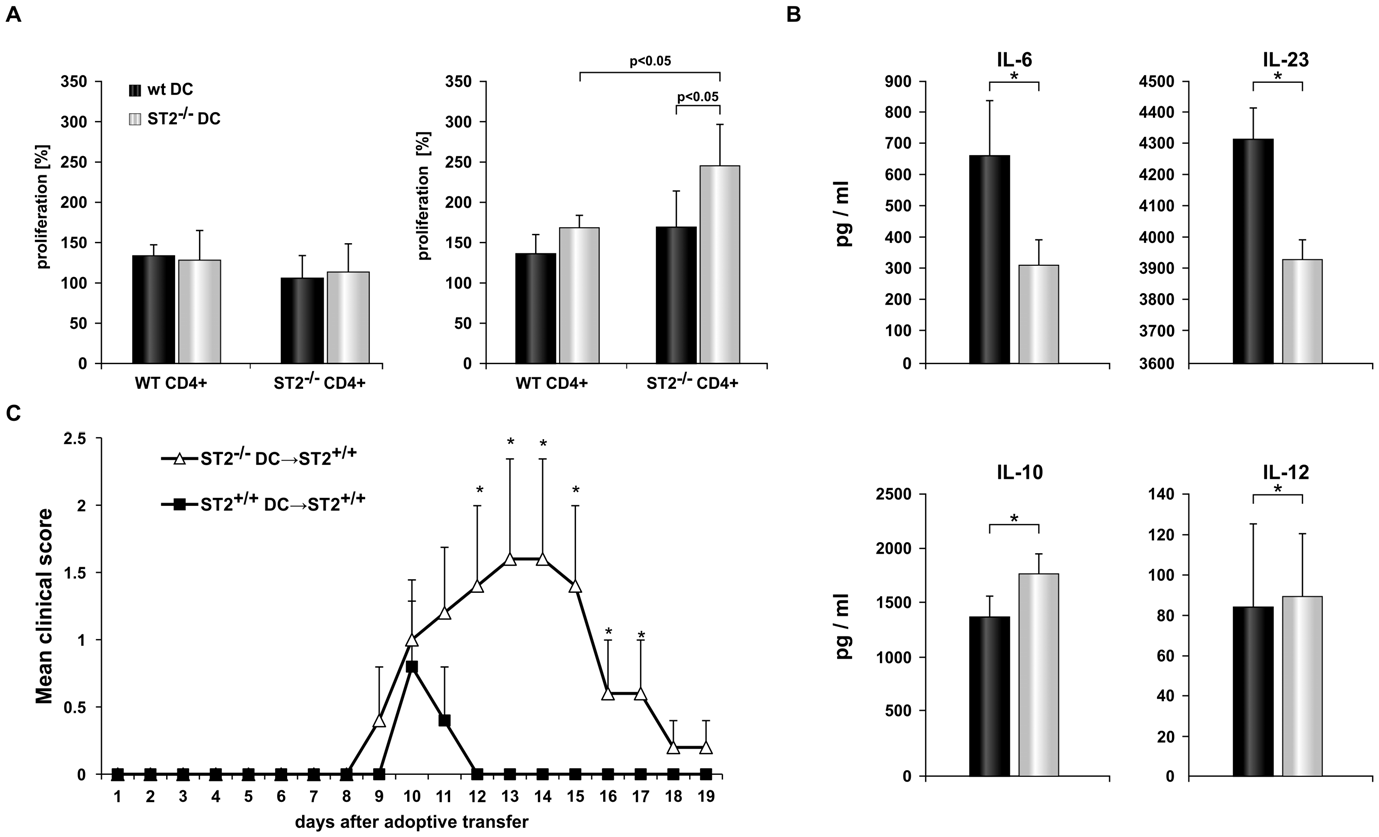
Absence of ST2 in dendritic cells enhances their APC capacity after EAE induction. Mice were immunized with MOG_35–55_/CFA and cells were isolated from lymph nodes and spleens. (A) Ability of ST2^−/−^ and WT dendritic cells isolated from spleen 8 days after immunization to affect proliferation of ST2^−/−^ CD4^+^ and WT CD4^+^ cells without or with MOG_35–55_ specific re-stimulation was determined by MTT assay. Proliferation is presented as percentage calculated in relation to proliferation of CD4^+^ cells only. (B) ELISA was performed to determine the production of IL-10, IL-6, IL-12 and IL-23 cytokines by dendritic cells isolated from spleens 9 days after immunization and stimulation with TLR1/2 agonist. Data are presented from 2 independent experiments as the mean+SD of representative experiment (n = 16 in both groups *P<0.05). (C) Dendritic cells were isolated from ST2^−/−^ and WT mice 4 and 8 days after MOG_35–55_ immunization. Active induction of EAE was performed on wild type BALB/c mice and after 4 and 8 days ST2^−/−^ or WT dendritic cells were given intravenously. Mice were clinically scored daily. Data are shown as mean+SD; 5 mice per group (*P<0.05). Statistical significance was tested by Student’s t-test.

**Figure 7:**
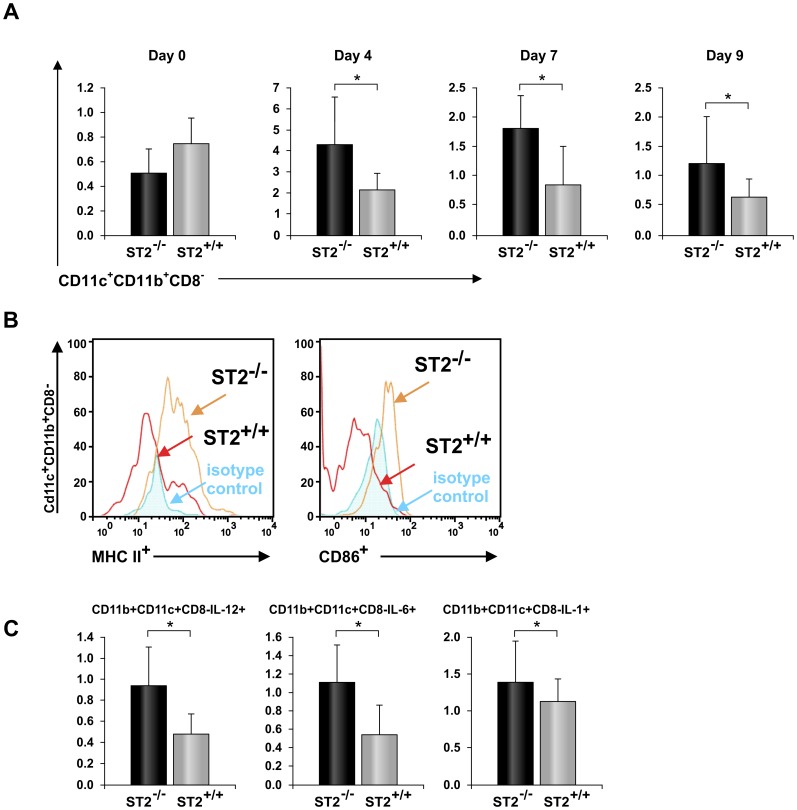
ST2 deletion leads to inflammatory phenotype of APC after EAE induction. Mononuclear cells were isolated from lymph nodes of ST2^−/−^ and WT BALB/c mice 4, 7 and 9 days after immunization and analyzed by flow cytometry. (A) Percentages of subpopulation of dendritic cells, CD11c^+^CD11b^+^CD8^−^, in the lymph nodes 4, 7 and 9 days after immunization are presented. (B) Expression of markers of activation, CD86 and MHC II, on dendritic cells was performed at day 4. Histograms were gated on CD11c^+^CD11b^+^CD8^−^ cells. (C) Percentages of CD11c^+^CD11b^+^CD8^−^ cells positive for IL-1, IL-12 and IL-6 in lymph nodes 4 days after immunization is presented. Data are presented from 3 independent experiments as the mean+SD of representative experiment (n = 23 in both groups). Statistical significance was tested by Student’s t-test.

### ST2 Deletion Facilitates Generation of Proinflammatory Cells in the Draining Lymph Nodes after MOG_35–55_ Immunization

To confirm that deletion of ST2 molecule in BALB/c mice affect generation of potentially encephalitogenic cells, we further analyzed CD4^+^ population collected from the draining inguinal lymph nodes 4, 7 and 9 days after MOG_35–55_ immunization. There was significantly higher (*p<0.001*) percentage of CD4^+^ lymphocytes containing IL-17, IFN-γ, TNF-α and GM-CSF in the draining lymph nodes of ST2^−/−^ BALB/c mice in comparison to BALB/c WT mice (Fig. 5A). There was only small percentage of CD4^+^ lymphocytes that contained IL-10 (data not shown) without significant difference between the groups. Significantly higher percentages of CD4^+^ cells expressing early marker of activation, CD69, were also found 4 and 7 days after immunization in draining lymph nodes of ST2^−/−^ BALB/c mice in comparison with BALB/c WT mice (Fig. 5B). Moreover higher percentages of CD4^+^ cells expressing chemokine receptors important for early influx into CNS, CCR6, CXCR5 and CXCR3 were found in lymph nodes of ST2^−/−^ BALB/c mice in the early phase of induction, concomitantly with their higher frequency in peripheral blood (Fig. 5C). Based on these findings it is possible that the higher susceptibility of ST2^−/−^ BALB/c mice to EAE could be derived from their ability to generate sufficient numbers of inflammatory encephalitogenic T cells.

### ST2 Deletion Affects Polarization of Antigen Presenting Cells

Results presented above suggested that ST2 deletion overcame resistance of BALB/c mice to EAE through polarization of helper T cells. ST2 molecule is expressed on antigen presenting cells and we next looked into the functional status of these cells.

We compared the capacity of dendritic cells isolated from ST2^−/−^ BALB/c mice or from BALB/c WT mice to support *in vitro* proliferation of CD4^+^ T cells isolated from MOG_35–55_ immunized mice. When CD4^+^ T cells isolated from BALB/c WT mice were cultured with ST2^−/−^ BALB/c, or BALB/c WT dendritic cells there was no significant proliferation (Fig. 6A). Only CD4^+^ cells derived from ST2^−/−^ mice cultured with ST2^−/−^ dendritic cells and restimulated with MOG_35–55_ had significant proliferation (Fig. 6A).

We next looked at the capacity of dendritic cells isolated from spleen to produce cytokines involved in Th1 and Th17 differentiation (IL-1, IL-6, IL-12 and IL-23), after TLR1/2 (Pam3CysSerLys4) stimulation. Splenic dendritic cells isolated from ST2^−/−^ BALB/c mice produced significantly higher amount of IL-6 and IL-23 while there was no difference in the production of IL-1 and IL-12. In contrast, IL-10 production was higher by dendritic cells isolated from BALB/c WT mice (p<0.05, Fig. 6B).

To directly demonstrate that ST2 deficiency in dendritic cells leads to the generation of encephalitogenic process adoptive transfer experiments were performed. BALB/c WT and ST2^−/−^ BALB/c mice were immunized with MOG_35–55_ peptide and after 4 and 8 days dendritic cells were isolated from spleens and draining lymph nodes. Dendritic cells (2×10^6^) were then given intravenously to BALB/c WT mice, 4 and 8 days after active EAE induction. Three of five BALB/c WT mice that received dendritic cells from ST2^−/−^ BALB/c mice developed clinical signs of EAE, one mouse was completely paralyzed (Fig. 6C). BALB/c WT mice that received WT dendritic cells developed significantly (p<0.05) milder clinical signs; two mice were only ataxic (Fig. 6C).

These results indicate that ST2 deficient dendritic cells facilitate development of encephalitogenic T cells that induce EAE.

In order to further analyze the characteristics of dendritic cells in ST2 deficient mice we looked at the presence of CD11c^+^ cells in draining lymph nodes in the inductive phase of the disease. Frequencies of dendritic cells in draining lymph nodes were similar in both groups (data not shown) but ST2^−/−^ BALB/c mice had significantly higher percentage of CD11c^+^CD11b^+^CD8^−^ inflammatory dendritic cells compared to BALB/c WT mice (Fig. 7A) 4, 7 and 9 days after immunization. There was no difference in percentages of CD11c^+^CD11b^+^CD8^−^ cells between naïve ST2^−/−^ BALB/c and BALB/c WT mice (Fig. 7A). Inflammatory phenotype of CD11c^+^CD11b^+^CD8^−^ cells was confirmed with further analyses. Higher expression of major histocompatibility complex class II and CD86 was found among CD11c^+^CD11b^+^CD8^−^ cells in lymph nodes of ST2^−/−^ BALB/c mice compared to these cells in draining lymph nodes of BALB/c WT mice (Fig. 7B). Also, production of Th1/Th17 cytokines in CD11c^+^CD11b^+^CD8^−^ cells was analyzed by flow cytometric analysis. On day 4 higher percentages of CD11c^+^CD11b^+^CD8^−^ cells containing IL-6, IL-1 and IL-12 was found in ST2^−/−^ BALB/c group of mice compared to BALB/c WT mice (Fig. 7C). This difference was significant (p<0.05) for cells containing IL-6 and IL-12.

These results suggest that ST2 deletion favor the expansion of inflammatory antigen presenting dendritic cells after antigen stimulation. It appears that expansion of encephalitogenic T helper cells in ST2^−/−^ BALB/c is associated with dominant polarization of APC toward inflammatory phenotype.

## Discussion

EAE is mediated by myelin specific T cells activated in the periphery that gain access to the CNS. These cells are reactivated in the CNS, produce various soluble mediators and recruit different inflammatory cells. This immunological events lead to tissue damage and targeting of its key elements may offer a number of advantages over currently available treatment strategies [35]. We have recently found that repeated administration of IL-33 may downregulate EAE in C57BL/6 mice while BALB/c mice after target disruption of ST2 molecule became susceptible to EAE [28].

The aim of this study was to analyse the events leading to the abrogation of the resistance to EAE in BALB/c mice.

Our results indicate that in contrast to WT mice, BALB/c mice with targeted disruption of ST2 gene after antigen stimulation develop highly inflammatory T helper cells able to enter CNS, produce inflammatory cytokines, disturb haematoencephalic barrier, enable influx of other immune cells in CNS and cause EAE in the absence of ST2 molecule in the target tissue.

Highly encephalitogenic potential of ST2^−/−^ lymphocytes was confirmed by adoptive transfer of these cells that induced the disease in inherently resistant BALB/c WT mice, as in ST2^−/−^ BALB/c mice (Fig. 4A). Moreover ST2^−/−^ BALB/c cells after MOG_35–55_ challenge have significantly higher expression of T-bet (Fig. 4C) that is critical for encephalitogenicity of both Th1 and Th17 myelin-specific T cells [36]. Lack of ST2 molecule in the inductive phase of the disease attenuates resistance of BALB/c mice to EAE.

Therefore we studied in more details CD4^+^ population in the lymph nodes early after encephalitogenic challenge. It appears that early after EAE induction (day 4 and 7) lymph nodes contain significantly higher percentage of CD4^+^ cells expressing early activation marker CD69 [37] in ST2^−/−^ BALB/c mice in comparison with BALB/c WT mice (Fig. 5B). Additionally mononuclear cells derived from the lymph node of ST2^−/−^ BALB/c mice 4 days after immunization contained significantly higher percentage of CD4^+^ cells expressing CCR6, CXCR3 and CXCR5 (Fig. 5C), chemokine receptors that appear to be important in auto reactive lymphocyte priming, emigration to the CNS and induction of EAE [38]–[44]. CCR6 is expressed on Th17 cells [45] and is required for the initial enter of T helper cells to the CNS through epithelial cells of the choroid plexus [39]. After helper T cells expressing CCR6 enter the CNS they trigger the entry of a second wave of T cells that now can enter into the CNS by crossing activated parenchymal vessels [39]. CXCR3 is mainly expressed on Th1 cells [46], and its blockade during EAE induced by adoptive transfer of myelin basic protein-activated T cells attenuated the disease [42]. Although CXCR5 is marker of T follicular helper cells [47], CXCR5 expressing T cells were found in EAE infiltrates of C57BL/6 [41]. Lymph node like structures that contain B lymphocytes are found in meninges of mice with EAE [47] and it is possible that CXCR5^+^ T lymphocytes in the CNS participate in the formation of these structures [48].

Possible mechanisms that lead to the development of encephalitogenic response include the following: a) ST2 deficiency on macrophages could promote M1 macrophage polarization that alters Th1/Th2 cytokine balance toward Th1/Th17 response as recently shown by us in C57BL/6 mice [28]; b) ST2 deficiency in innate cells type 2, nuocytes, or natural helper T cells might alter the natural Th2 bias in BALB/c mice [49], or c) lack of ST2 molecule on antigen presenting cells (dendritic cells) leads to inflammatory cytokine production and development of encephalitogenic T helper cells.

Since the development of T helper phenotype mostly depends on function of APC [50] we focused on the effects of ST2 molecule on APC during EAE. To this end we analyzed the capacity of ST2^−/−^ and WT dendritic cells to restimulate primed CD4^+^ T lymphocytes. Deletion of ST2 molecule amplifies the ability of dendritic cells to stimulate proliferation of antigen specific CD4^+^ cells (Fig. 6A). These data suggested that in the absence of IL-33/ST2 signaling dendritic cells appear to be more efficient in restimulating encephalitogenic cells. Further, dendritic cells isolated from ST2^−/−^ BALB/c mice immunized with MOG_35–55_ peptide, after *in vitro* stimulation with TLR1/2 agonist, produced higher amount of IL-6 and IL-23 and lower amount of IL-10 in comparison with dendritic cells from BALB/c WT mice (Fig. 6B). Inflammatory ST2^−/−^ dendritic cells could induce polarization oh T helper cells toward Th1/Th17. These results that ST2^−/−^ dendritic cells produce more inflammatory cytokines are in apparent contrast with previous findings of Besnard et al. that IL-33 stimulation enhances production of IL-6, IL-1β and TNF in dendritic cells [51]. However these IL-33 stimulated dendritic cells were not found to produce IL-12 and therefore directed immune response toward Th2 type. Further, results of our study are in agreement with findings that ST2 molecule is constitutively expressed on myeloid cells where it has a role as negative regulator of TLR2 and TLR4 signaling directly decreasing the production of TNF-α, IL-6 and IL-12 in these cells [52], [53].

The analysis of phenotypic differences between dendritic cells derived early after disease induction (day 4) showed that the percentage of the CD11c^+^ cells in the lymph nodes was similar. Although there was no difference in the percentages of CD11c^+^ cells, immunized lymph nodes of ST2^−/−^ mice contained higher frequencies of CD11c^+^CD11b^+^CD8^−^ cells (Fig. 7A). A inflammatory dendritic cells CD11c^+^CD11b^+^ that migrate to draining lymph nodes after virus infection and immunization with CFA (Th1 stimulus) produce abundant IL-12 and stimulate IFN-γ production in T lymphocytes [54]. However there are also several reports that CD11c^+^CD11b^+^ dendritic cells are immunosuppressive cells and play important role in tolerance induction. We found that draining lymph nodes of ST2^−/−^ BALB/c mice, after immunization with MOG_35–55_ peptide in CFA, contained higher percentage of CD11c^+^CD11b^+^ CD8^−^ dendritic cells and confirmed inflammatory phenotype of these cells, while lymph nodes of naïve mice had similar percentages of these cells. They also had higher expression of markers of activation, CD86 and MHC II (Fig. 7B) and significantly higher percentage of these cells in draining lymph nodes of ST2^−/−^ BALB/c mice contained inflammatory cytokines IL-12 and IL-6 (Fig. 7C) compared to CD11c^+^CD11b^+^ CD8^−^ cells in lymph nodes of BALB/c WT mice. It is of interest to note that 1,25-dihydroxyvitamin D3 dependent human tolerogenic dendritic cells are also found to maintenance a lower expression of CD86, MHC II as seen in WT BALB/c mice [55].

Effects of ST2 deletion on dendritic cells in development of EAE is confirmed with passive transfer experiment: MOG_35–55_ immunized BALB/c WT mice developed EAE after intravenous administration of dendritic cells isolated from immunized ST2^−/−^ BALB/c mice (Fig. 6C).

Data presented here and report of Jiang et al. [28] are in apparent contrast with findings of Li et al. [27] that IL-33 administration in the inductive phase of EAE aggravates the disease, while anti-IL-33 antibody significantly inhibits the onset and severity of EAE by reducing MOG_35–55_-stmulated IFN-γ and IL-17 production. In the natural course of the disease, IL-33 is released in its active form only upon necrosis, and necrotic tissue destruction in CNS only becomes apparent late in EAE. Besides anti-IL-33 antibody used in the study of Li et al [27] is polyclonal and its neutralizing capacity is not demonstrated. Also, as it was shown for other cytokine/receptor pairs [56], is possible that ST2 could be a component of receptors for another ligand(s) besides IL-33, or that IL-33 may bind to other receptors besides ST2. It could be also assumed that exogenous IL-33 given in the inductive phase of EAE potentiates production of inflammatory cytokines in myeloid cells stimulated with adjuvant while therapeutic application of IL-33 has anti-inflammatory effects.

In summing up we have shown that inherent resistance to EAE induction in an inbreed strain of mice may depend on IL-33/ST2 signaling in the initial phase of encephalitogenic process and is directly related to the induction of inflammatory phenotype of antigen presenting cells during initial priming of encephalitogenic cells.
